# Coronary Artery Disease in Very Young Women: Risk Factors and Prognostic Insights from Extended Follow-Up †

**DOI:** 10.3390/jcdd12020034

**Published:** 2025-01-21

**Authors:** Samuel Pintos-Rodríguez, Víctor Alfonso Jiménez Díaz, César Veiga, Carlos Martínez García, Francisco Caamaño Isorna, Andrés Íñiguez Romo, Pablo Juan-Salvadores

**Affiliations:** 1 Cardiovascular Research Group, Galicia Sur Health Research Institute (IIS Galicia Sur), SERGAS-UVIGO, 36312 Vigo, Spain; samuel.pintos@iisgaliciasur.es (S.P.-R.); victor.alfonso.jimenez.diaz@sergas.es (V.A.J.D.); cesar.veiga@iisgaliciasur.es (C.V.); carlos.martinez@iisgaliciasur.es (C.M.G.); andres.iniguez.romo@sergas.es (A.Í.R.); 2 Cardiovascular Research Unit, Cardiology Department, Hospital Álvaro Cunqueiro, University Hospital of Vigo, 36312 Vigo, Spain; 3 Department of Psychiatry, Radiology, Public Health, Nursing and Medicine, IDIS University of Santiago de Compostela, 15706 Santiago de Compostela, Spain; francisco.caamano@usc.es; 4 CIBER of Epidemiology and Public Health (CIBERESP), Instituto de Salud Carlos III, 28029 Madrid, Spain; 5 Cardiology Department, Complexo Hospitalario Universitario de Vigo (SERGAS), Álvaro Cunqueiro Hospital, 36312 Vigo, Spain; 6 CIBER of Cardiovascular Disease (CIBERCV), Instituto de Salud Carlos III, 28029 Madrid, Spain

**Keywords:** coronary artery disease, young women, percutaneous coronary intervention, Castelli index, depression, secondary prevention

## Abstract

Coronary artery disease (CAD) is usually associated with the elderly, but an increase in its incidence has been recently reported among young people, including very young women. The aim of this study is to assess the associations between different clinical variables and the risk of early CAD and occurrence of major adverse cardiovascular events (MACEs) during follow-up. Our cohort consists of women ≤40 years referred for coronary angiography due to suspicion of CAD; a nested case–control study was conducted among these patients. In total, 19,321 coronary angiographies were performed between 2006 and 2015, of which 2.6% were in patients ≤40 years old; 52 women were finally included. Family history of CAD was strongly associated with the early onset of the disease [OR 5.94, 95%CI (1.13–31.15); *p* = 0.035] in young women. The incidence of MACE was also associated with depression [HR 8.20 95%CI (1.03–65.17); *p* = 0.047] and Castelli Index [HR 11.49, 95%CI (1.40–94.51); *p* = 0.023]. Primary prevention focused on genetic analysis for high-risk women with a family history of CAD and secondary prevention, targeting a better cholesterol management and mental health assistance must be considered.

## 1. Introduction

Coronary artery disease (CAD) is one of the most common cardiovascular diseases and usually appears after the sixth decade of life, with slightly differences in the age of presentation between sexes [1]. However, some populations experience this disease prematurely [2]. The prevalence of CAD reported in very young people ranges between 1 and 16% [3,4,5,6,7], with acute coronary syndrome (ACS) being its most common presentation. Among them, very young women are a sub-population group of particular interest [8,9,10].

The association of CAD with male sex led to an underrepresentation of women in studies addressing this topic, even though young women have distinct risk profiles and clinical presentations [11]. Young women usually have a higher prevalence of comorbidities and cardiovascular and psychosocial risk factors that could predispose them, in combination with other sex-specific risk factors, such as history of adverse pregnancy outcomes and use of combined oral contraception, to present atypical symptoms and worst clinical risk scores [11,12,13]. This suggests that we can consider young women as a population of particular interest due to the multifactorial and complex interrelationships that lead to the onset of the disease on them [13]. Also, these young patients, despite being a small proportion, represent a significant economic and health care burden because they become chronic patients at very early stages of life and developed more aggressive forms of the disease too [14].

Nevertheless, studies addressing CAD on these population are scarce, and most of them focused on postmenopausal women or used cut-off points for age that vary widely among them, hindering the comparability of their results either clinically or in long-term follow-up [11,12,13,15,16,17]. Although an association with traditional risk factors exists [5,11,13,15,18,19,20,21], the attributable increase in the risk is not ascertained to be the same as in older or male populations due to biological and physiological factors or age-related comorbidities. Additionally, few studies evaluated the prognosis of subjects below ≤40 years of age with cardiovascular risk factors and normal coronary arteries or non-significant coronary stenosis evaluated using angiography [17,22,23,24]. Even fewer studies put the scope in young women with these cardiovascular characteristics.

Therefore, the aim of this study is to provide insight in the study of risk factors related to the onset of CAD in very young women, as well as evaluating the risk of developing a major adverse cardiovascular event (MACE) during follow up, opening the scope for future studies in this area.

## 2. Methods

### 2.1. Design and Study Population

This study is a single-centre, retrospective, nested case–control study in a cohort of patients aged ≤ 40 years old undergoing coronary angiography due to clinical suspicion (electrocardiographic changes, biomarkers of myocardial injury, or a positive ischemia stress test) of CAD, including acute coronary syndrome (ACS), stable angina, or silent ischemia. This article is a revised and expanded version of a paper previously published [25].

**Figure 1:**
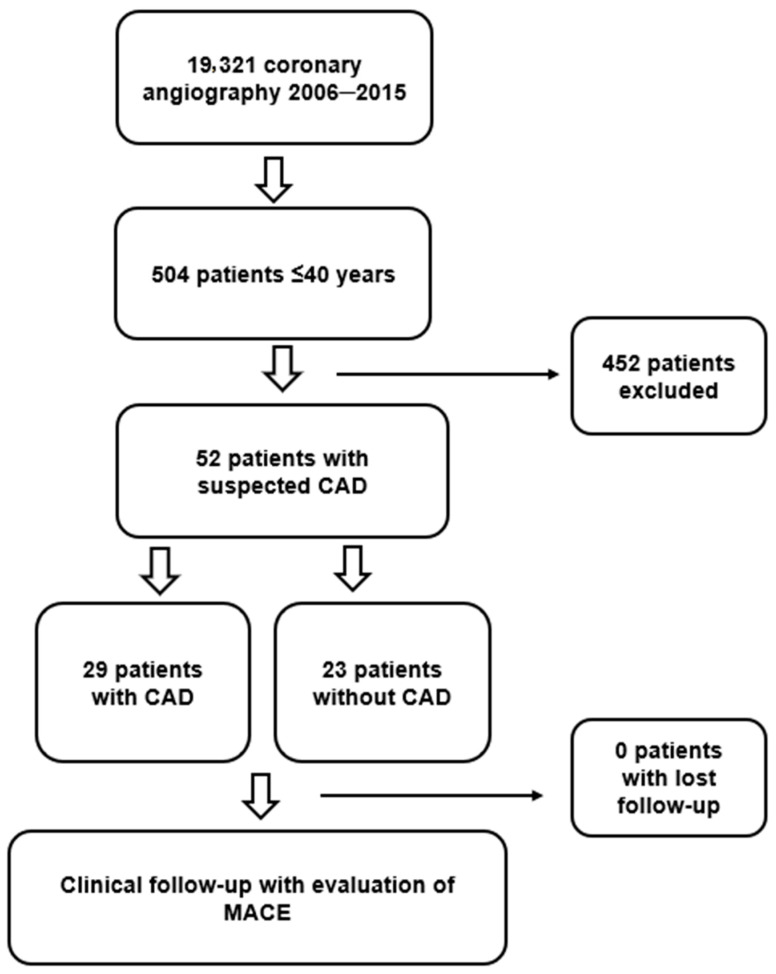
Flow chart diagram of the study population.

From 1 January 2006 to 31 December 2015, a total of 19,321 coronary angiograms were performed in our hospital, and 504 (2.6%) were in patients ≤40 years old. After exclusions of males and women presenting exclusion criteria, 52 women were ultimately included in the study (Figure 1).

Cases and controls were defined as follows:

- Cases: Women aged between 18 and 40 years referred for coronary angiography due to suspicion of CAD based on signs or symptoms or a positive ischemia stress test. They must present at least one angiographically significant coronary stenosis (≥75% of the luminal diameter determined visually or ≥50% by quantitative coronary analysis; in the left main, stenosis ≥ 50% was considered a significant disease) or any positive invasive fractional flow reserve test in one or more epicardial vessels.
- Controls: Women aged between 18 and 40 years referred for coronary angiography due to suspicion of CAD based on signs or symptoms or a positive ischemia stress test but with non-diagnostic of CAD.

The exclusion criteria were the same for cases and controls, they included presenting a prior coronary revascularization (percutaneous or surgical) or referral for coronary angiography for any other non-ischemic cause (pre-transplant, pulmonary hypertension, structural disease, among others).

In this study, we also assessed the risk of developing MACE during follow-up; both cases and controls were followed for evaluating possible differences between them. The median follow-up time was 5 years, ranging from a minimum of 1 year to a maximum of 9.

The age of inclusion was restricted to ≤40 years following the findings of the Coronary Artery Risk Development in Young Adults Study (CARDIA), as they reported that the risk of suffering a coronary ischemic event increases significantly over 40 years. A difference in prevalence of 13.3% vs. 5.5% was described when comparing groups of patients aged 40–45 years and 33–39 years, respectively [26]. These findings show that something could change around 40 s, which is in agreement with our objective of describing the most important factors related to an early onset of the disease; we think that this cut-off point fits properly with the objectives previously described.

### 2.2. Data Collection and Definition of Variables

The study population came from a tertiary hospital, a reference centre for cardiovascular disease. The information was obtained through the specific database of the Interventional Cardiology Unit, disaggregated by any personal data. The follow-up was conducted via a database analysis, which was updated using electronic medical records.

Qualitative variables were defined as follows: arterial hypertension (defined as a previous diagnosis recorded in the clinical history or by the use of hypertensive medication), diabetes mellitus (having a previous diagnosis or by the use of hypoglycaemic medication), dyslipidaemia (based on previous diagnosis or use of lipid-lowering medication), body mass index (BMI) (classified as normal weight < 25, overweight 25–29, and obesity ≥ 30), smoking (current or former smoker), drug consumption (active consumption of toxic substances recognised by the patient or by laboratory tests), family history of coronary artery disease (defined as direct family history of cardiovascular disease before age 55 for men, and before age 65 for women), and depression (based on previous diagnosis or consumption of antidepressant medication). Quantitative variables were defined, as these related to the lipid profile of the patients or resulting from laboratory analysis, such as the following: total cholesterol, HDL-C, LDL-C, triglycerides, creatinine, glucose or residual cholesterol, and atherogenic indexes like Castelli index, Kannel index, triglycerides/HDL index, triglycerides/glucose index were also recorded or calculated from primary values.

The composite variable MACE included death, myocardial infarction, stroke, and new coronary revascularization (shall be collected the occurrence of one of these events for the first time in each patient).

### 2.3. Ethical and Legal Aspects

The investigators participating in this study followed the applicable ethical and legal standards. This study was approved by the Regional Research Ethics Committee with registration code 2015/506.

### 2.4. Statistical Analysis

Descriptive statistics are reported as mean ± standard deviation (SD) or median with interquartile range (IQR) for continuous variables and numbers and percentages for categorical variables. A bivariable analysis was performed to detect significant differences between cases and controls for the primary variables using a Fisher exact test, chi^2^ test, and Student’s *t* test, as appropriate. To determine the contribution of different factors to the risk of developing CAD, a multivariate logistic regression analysis was performed using a Wald backward method. The cardiovascular risk factors analysed are given as odds ratio (OR) with their 95% confidence intervals (95%CI). Kaplan–Meier curves and the log-rank test were used to compare time to MACE between the two groups of patients. To identify the factors associated with MACE, a binary logistic regression analysis using the backward method of Wald, calculating the hazard ratios (HR) and 95% CIs. For the data analysis, IBM SPSS Statistics for Windows (version 26.0; IBM Corp.: Armonk, NY, USA, 2019) was used.

## 3. Results

## Study Characteristics

The clinical characteristics of the patients at admission are shown in Table 1. Age had a median of 35 years and an IQR of 26–40 years; no differences were observed between groups (*p* = 0.879). Among cases, a higher proportion of smokers was present (*p* = 0.014), and having a family history of coronary disease was also more common (*p* = 0.008). In the multivariate logistic regression analysis, only the family history of CAD showed a significant association with the onset of CAD in young women [OR 5.94 (CI 95% 1.13–31.15); *p* = 0.035].

Among cases, the vast majority presented with one vessel with a significant stenosis (n = 21; 72.4%), although 8 (27.6%) had a multivessel lesion. The median baseline stenosis was 94.2 ± 8.2%, and only one presented a calcified lesion, with AHA classification type C 9 (47.4) In most of these women, the affected coronary artery was the left anterior descending (LAD) and branches (n = 16; 55.2%). One stent was implanted in 27 (93.1%) of the cases, and the overall final stenosis was 21.4 ± 41.8.

The pharmacological treatment of both groups is shown in Table 2. At hospital admission, only the consumption of acetylsalicylic acid (ASA) was statistically different between groups (*p* = 0.040), and no differences were observed related to other compounds. At discharge, significant differences were found between them on statins, angiotensin-converting enzyme inhibitors (ACE-I), β-blockers, and ASA and P2Y12 inhibitors; all of them were more prescribed in the case group.

**Figure 2:**
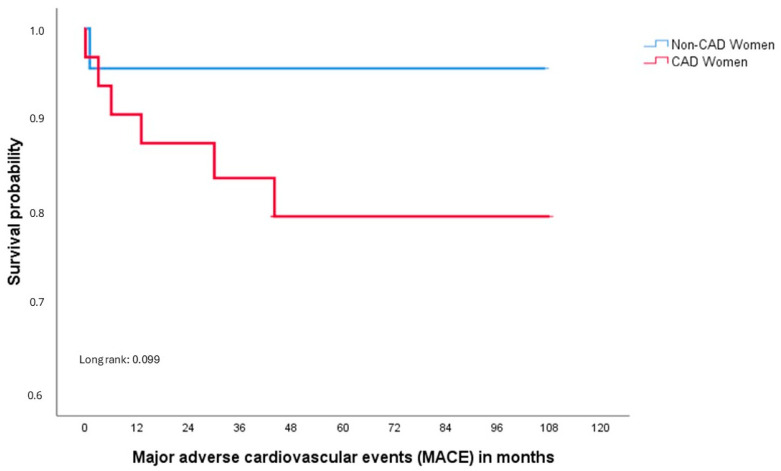
Clinical outcomes of case and control groups at the time of the first MACE.

The most referred symptom at admission was chest pain in both groups. Although ACS was the most common indication for coronary angiography among cases and controls, significant differences were observed between them (*p* = 0.005). The median follow-up time was 5 years, ranging from a 1-year minimum to a 9-year maximum, and both the follow-up time and days of hospitalization were greater in the control group compared to the cases; however, it did not reach statistical significance. During the follow-up, 7 MACEs occurred, one in the control group and six in the case group; both the event in controls and most of outcomes (n = 3) among cases were due to new coronary revascularizations. As was expected, the case group showed a higher incidence of MACE during the follow-up (*p* = 0.086) (Table 3). The Kaplan–Meier’s curve for MACE is shown in Figure 2, and no statistical differences were observed between groups (*p* = 0.099).

On the bivariate analysis, depression, glucose, high levels of triglycerides, Castelli index, and Kannel index were significantly different between the groups (Table 4). Despite that, in the binary logistic regression analysis, only depression [HR 8.20 (CI 95% 1.03–65.17); *p* = 0.047] and Castelli index [HR 11.49 (CI 95% 1.40–94.51); *p* = 0.023] remain significant, showing association with an increased risk of MACE during follow-up.

## 4. Discussion

This study is the first in women ≤40 years with such a long follow-up, so its results provide useful information in different ways. Firstly, in our cohort, the family history of CAD is the most closely related factor with the onset of early CAD in very young women. Secondly, a higher proportion of heavy smoking and a lower proportion of classical risk factors as hypertension or diabetes mellitus were observed. Thirdly, during follow-up, depression and Castelli index were significantly associated with an increased risk of presenting MACE. Therefore, these findings show the possible existence of a hereditary component related to the onset of the disease and the huge impact that mental health and a proper cholesterol control could have on the course of the disease on these young women.

### 4.1. Factors Associated with Early CAD

The onset of early CAD in young women seems to be associated with the interaction of different risk factors, either modifiable or non-modifiable, which give this disease a multifactorial aetiology [11,13,15]. Among these non-modifiable factors, the family history of CAD was identified as the most influencing factor in our study, shedding light on the possible existence of genetic factors related to the development of the disease. In our cohort, this could be corroborated because over 40% of the cases had a family history of CAD. This assumption can be supported by the findings of previous studies that reported high prevalence of family history of CAD among young CAD-adults [12,13,17,20]. A family history of premature atherosclerosis in both first- and second-degree relatives and a high polygenic score were both identified as a possible origin of this association [15,21].

Prior studies have identified smoking as one of the main factors related to the onset of early CAD in young patients [12,17,24,27]. The prevalence of smoking in those studies ranged from 49.7% to 95% [15,20,21], while in our study, it was of 89.7% among cases, highlighting the worryingly high proportion of active smoking in our cohort. Despite that, smoking was not associated with the onset of early CAD in our study. We suggest that it could be because of the also high proportion of active smokers among the controls (60.9%), who were drawn from the same population as cases (patients who referred for coronary angiography due to suspicion of CAD based on signs or symptoms, or a positive ischemia stress test) and not from the general population. The huge impact smoking has on cardiovascular health is well known. Either active or passive smoking promotes endothelial disfunction, activation of proinflammatory pathways, and induces coronary vasoconstriction, which, in combination, lead to initiation and progression of the atherothrombotic disease [13,27], and very young women are not exempt from that. For example, Lu et al. reported that current smoking was the second factor mostly associated with the onset of early CAD in women <55 years old, with a population-attributable fraction of 38.9% on their cohort [15]. These findings are supported by a meta-analysis which concluded that young women ≤50 years who smoked either actively or passively were prone to suffer ACS in their life [13].

The role of other widely known risk factors, such as hypertension, diabetes, obesity, and drug abuse, as triggering factors of CAD in young patients have been previously evaluated, giving mixed results [4,11,16,17,18,24,28,29]. Our study fails to demonstrate a significant relationship between them and the presence of CAD, due to their low incidence in our cohort and the chronic pattern of most of them, showing their effects on health after a long period of time. For example, only one person had diabetes, and five were drugs users. Our rate consumption (9.6%) agrees with a published study in which the prevalence of cannabis smoking was of 11.0% and 8.9% among young women (≤50 years) with STEMI and non-STEMI, respectively [12]. Based on these studies, we think that cardiovascular risk factors differ among extremely young, young, and elderly patients with ACS [17,24], and young women are not an exception to that. Reaching a consensus on the cut-off age to which women who develop CAD are considered young is mandatory to provide homogeneity in future studies; this would allow for a better comparability of the results obtained and identification of risk factors associated with the onset and prognosis of the disease in this population.

The pharmacological treatments provided to these patients have different objectives. Prior to hospital admission, it focused on primary prevention of the disease by controlling the risk factors related to its onset. At this point, we observed that only 1 (1.9%) woman was at statin treatment despite 16 (30.8%) present dyslipidaemias; this figure is even worse than that observed in a previous study, where although 26.4% of patients had dyslipidaemia, only 8.2% were under treatment [12]. At hospital discharge, the pharmacological treatment had as its main objective reducing anginal symptoms and preventing adverse events, both pillars of secondary prevention. Significant differences have been found between study groups, related to their different clinical diagnoses established after coronary angiography. Due to the increased expectancy of life in the general population and the improvement of health-related quality of life on these patients, an appropriate therapeutic adherence is crucial, especially due to the low-risk perception of the disease after an initial event [30]; for example, in the study of Papathanasiou et al., only 65% of male patients continued to take statin over the follow-up period [17]. One of the main reasons of this underestimation of the risk could be due to underrepresentation of the youngers (especially women) in clinical studies concerning CAD-related risk factors and outcomes [21]. The evidence generated is clear, as poor adherence increases the risk of suffering an adverse event in these young cohorts where the disease presents in such aggressive forms [31]. An example of that is the relatively high proportion of women who experienced a MACE during follow-up in our study (1 of each 5), despite improvements in revascularization techniques, and that majority of CAD cases correspond to non-STEMI rather than STEMI. Given that we are treating these young women like their older counterparts, we suggest using all the available therapeutics to avoid relapses, like PCSK9 inhibitors or new pharmacological treatments which target lipoprotein-A (Lp-a) to improve their long-term outcomes. Although Lp (a) is widely known as an important cardiovascular risk factor, the lack of data on this very young population means that further studies are needed to assess whether these therapeutics can improve their cardiovascular health in the short or long term. These therapies should ideally be initiated as early as possible, preferably during hospitalization, to maximise their benefits and ensure better adherence to long-term treatment plans.

### 4.2. Follow Up

The differences observed on hospital stay and follow-up could be associated with the fact that, once the presence of CAD is ruled out, more complementary tests should be taken to assess other possible diagnosis. The most common event during follow-up was presenting a new coronary revascularization, similar to other studies [17,32]. The mortality was low in our cohort, with only one death corresponding to a case. The early care programme for myocardial infarction, the subtype of CAD presented, and the youth of our cohort could be some of the reasons [33]. The mortality found in our study of 3.4% is in concordance with the previous literature, which ranged it between 2.0% and 8.5% [16,17,18,21,24]. Jason et al. also reported a 31% mortality at 15 years [32], reflecting the importance of providing an individualised and high-quality secondary prevention in this population, as they will live for the rest of their lives with a very aggressive disease.

In our study, the factors which showed a more significant association with the occurrence of MACE during follow-up were non-traditional cardiovascular risk factors, such as depression and an atherogenic index underused in very young patients of cardiovascular studies, the Castelli index. Other factors whose influence is widely known, like diabetes mellitus, could not have been evaluated in this study due to lack of participants with the disease. Focusing efforts on an accurate control of cholesterol levels using atherogenic indexes for a better identification of patients at risk, along with all the available therapeutics, such as iPCSK9 or new pharmacological treatments which target Lp-a, could help to achieve a more effective secondary prevention among these patients. Cholesterol is a previously known risk factor, and the use of these indexes could allow us to easily classify these patients, more than an isolated value of abnormal HDL or LDL cholesterol.

Depressive symptoms occur in one in every three patients with AMI and had been previously associated with poorer long-term outcomes, as its incidence in young adults with AMI is increasing [21,34]. Young women with AMI presented with higher levels of psychosocial risk factors, such as depression, stress, poorer physical and mental health status, and lower quality of life at the time of the event in comparison to men [11,29,34]; Lu et al. also reported that depression’s PAF among women is 3-fold more than in men (25.5 vs. 8.7%), and this association remains after adjustment, suggesting that both psychological and socioeconomic risk factors play an important role in the onset and prognosis of AMI on them [15]. In our study, the prevalence of depression was of 17.3%, which is lower than previously reported [12], as in other studies, nearly half of the women presented with a previous diagnosis of depression or were under antidepressant treatment at onset [11,29]. Although the nature and causality of the mechanisms that might explain this association have not been elucidated, prior studies have hypothesised that depression may increase women’s risk of cardiovascular disease by different biological (elevating atherosclerotic and inflammatory biomarkers, reducing pulse rate variability, and increased platelet reactivity) and behavioural (low social support, smoking, alcohol abuse, obesity, poor medication adherence) mechanisms [11,34,35]. These findings highlight the need for new sex-specific strategies focused on gender-related characteristics, which will provide young women with CAD a better prognosis [15]. Presenting depressive symptoms at the same time as having CAD increases both mortality and morbidity due to CAD and may also negatively affect patients’ recovery and limit their daily function [21,34,35].

The results provided by this study could be very insightful in terms of the prevention of cardiovascular diseases and promotion of overall health among young women, both key points in current public health policies. The high risk of re-infarction or other MACE related to the main event, high amounts of medication for life, and the almost high consumption of health services prove that this disease not only has a high impact on the quality of life of the patients, but it also has huge costs for the social and health care systems. That is why an early detection of risk factors related to the onset of CAD and an appropriate set of preventive actions to control them are necessary. Novel atherogenic indexes, such as Castelli’s index, could help practitioners to better classify patients based on their atherogenic risk, while establishing fast-track access routes to psychological support consultations could help young women with depression to better manage their disease and diminished the effects of CAD on their long-term health. Secondary prevention in young women with CAD requires a comprehensive approach that includes novel antiplatelet strategies based on platelet function testing or genetic markers if needed, along with a better management of modifiable risk factors through lipid-lowering therapies and lifestyle interventions focused on mental health, regular physical activity, and a better dietary control.

This study has several limitations. Firstly, due to the nature of our study, selection biases cannot be ruled out. Secondly, due to the small number of cases, the CIs were too wide, but despite that, we achieved statistical significance. Thirdly, despite lipoprotein (a) being widely known as an independent cardiovascular risk factor, we do not have measurements of its values; the inclusion of lipoprotein (a) in future studies could help to refine the observed associations. Fourthly, our analysis did not differentiate between patients receiving ASA as monotherapy or in combination with IP2Y12 due to the small proportion of patients at monotherapy. As strengths, this is one of the first studies which assessed the risk of developing CAD in a population of very young women, paving the way for future studies with bigger databases and statistical power to better refine our results and provide new information about other classical or novel risk factors. The finding of atherogenic indexes as a useful tool is also a major contribution to our work, which will allow for a better classification of patients at risk. Lastly, the identification of depression as one of the most important factors related with MACE during follow-up puts the scope on the importance of mental health and provides an accurate psychological support to these patients.

## 5. Conclusions

In our study, the family history of CAD was found to be the most significant factor associated with the onset of CAD in women ≤40 years old. In addition, high values in the Castelli index and presenting depression were significantly associated with an increased risk of MACE during follow-up. A detailed genetic analysis on high-risk women with a family history of CAD and other cardiovascular risk factors and promotion of health focused on a better control of the cholesterol levels and mental health should be considered.

## Figures and Tables

**Table 1:** Clinical characteristics of cases and matched controls (CAD vs. non-CAD).

Characteristic	Overall (n = 52)	Non-CAD Women (n = 23)	Women with CAD (n = 29)	*p*-Value
Age, median (IQR)	35 (26–40)	35 (26–40)	35 (26–40)	0.879
Family History of CAD; n (%)	14 (26.9)	2 (8.7)	12 (41.4)	0.008 *
Follow-up time (years)	4.9 ± 2.3	5.4 ± 2.4	4.5 ± 2.2	0.144
Hospitalization days	6.9 ± 7.4	7.8 ± 11.0	6.2 ± 3.1	0.522
**Comorbidities; n (%)**				
Hypertension	9 (17.3)	4 (17.4)	5 (17.2)	0.989
Diabetes	1 (1.9)	0 (0)	1 (3.4)	0.369
Current smoker	40 (76.9)	14 (60.9)	26 (89.7)	0.014 *
BMI ≥ 30 kg/m^2^	14 (26.9)	7 (30.4)	7 (24.1)	0.611
Dyslipidaemia	16 (30.8)	7 (30.4)	9 (31.0)	0.963
Drug consumption	5 (9.6)	2 (8.7)	3 (10.3)	0.841
Depression	9 (17.3)	5 (21.7)	4 (13.8)	0.452
**Lipid levels and Atherogenic Indexes; n (%)**				
Total cholesterol	190.8 ± 50.6	188.7 ± 40.8	192.4 ± 57.8	0.274
HDL-C	45.8 ± 14.8	47.4 ± 14.5	44.9 ± 15.2	0.807
LDL-C	120.6 ± 46.2	125.8 ± 34.7	117.5 ± 52.1	0.457
Triglycerides	144.0 ± 117.6	142.2 ± 78.4	145.4 ± 143.2	0.515
Creatinine	0.75 ± 0.17	0.79 ± 0.17	0.72 ± 0.16	0.508
Glucose	105.2 ± 30.5	101.9 ± 29.3	107.7 ± 31.7	0.503
Castelli Index	4.6 ± 1.8	4.6 ± 1.71	4.7 ± 1.87	0.873
Kannel Index	2.9 ± 1.4	3.0 ± 1.4	2.9 ± 1.5	0.843
TGL/HDL Index	3.8 ± 4.0	3.5 ± 2.3	4.02 ± 4.77	0.325
Residual Cholesterol	26.8 ± 14.1	28.3 ± 15.0	25.9 ± 13.7	0.582
TGL/Glucose Index	8.71 ± 0.68	8.68 ± 0.60	8.73 ± 0.75	0.673
**Admission Symptoms**				
Chest pain	46 (88.5)	19 (82.7)	27 (93.1)	0.239
Dyspnoea	5 (9.6)	1 (4.3)	4 (13.8)	0.251
Shock	5 (9.6)	3 (13.0)	2 (6.9)	0.455
LVEF (%)	54.7 ± 11.7	52.5 ± 15.3	56.3 ± 7.9	0.313
**Coronary angiography indication**				
ACS	41 (78.8)	14 (60.9)	27 (93.1)	0.005 *
STEMI	18 (34.6)	4 (17.4)	14 (48.3)	
NSTEMI	23 (44.2)	10 (43.5)	13 (44.8)	
Stable angina	11 (21.2)	9 (39.1)	2 (6.9)	

CAD = coronary artery disease; IQR = interquartile range; BMI = body mass index; HDL = high-density lipoproteins; LDL = low-density lipoproteins; TGL = triglycerides; ACS = acute coronary syndrome; STEMI = ST-elevation myocardial infarction; NSTEMI = non-ST-elevation myocardial infarction. * *p*-value ≤ 0.005.

**Table 2:** Pharmacological treatment at hospital admission and at discharge.

Characteristic	Overall (n = 52)	Non-CAD Women (n = 23)	Women with CAD (n = 29)	*p*-Value
**Pharmacological treatment at hospital admission.**				
Statins	1 (1.9)	1 (4.5)	0 (0)	0.246
CCB	2 (3.8)	1 (4.5)	1 (3.4)	0.842
ARBs	1 (1.9)	0 (0)	1 (3.4)	0.379
ACE inhibitors	3 (5.8)	1 (4.5)	2 (6.9)	0.724
Beta Blockers	2 (3.8)	2 (9.1)	0 (0)	0.098
Anticoagulants	1 (1.9)	0 (0)	1 (3.4)	0.379
ASA	3 (5.8)	3 (13.6)	0 (0)	0.040 *
P2Y12 Inhibitors	1 (1.9)	1 (4.5)	0 (0)	0.246
**Pharmacological treatment at discharge.**				
Statins	27 (54.0)	4 (18.2)	23 (82.1)	<0.001 *
CCB	4 (8.0)	2 (9.1)	2 (7.1)	0.801
ARBs	1 (2.0)	0 (0)	1 (3.6)	0.371
ACE-I	12 (24.0)	1 (4.5)	11 (39.3)	0.004 *
Beta Blockers	31 (62.0)	6 (27.3)	25 (89.3)	<0.001 *
Anticoagulants	0 (0)	0 (0)	0 (0)	-
ASA	38 (76.0)	10 (45.5)	28 (100.0)	<0.001 *
P2Y12 Inhibitors	26 (52.0)	2 (91.1)	24 (85.7)	<0.001 *
Oral contraceptives	4 (8.0)	2 (9.1)	2 (9.1)	0.801

CAD = coronary artery disease; CCB = calcium channel blockers; ARBs = angiotensin II receptor blockers; ACE-I = angiotensin-converting enzyme inhibitors; ASA = acetylsalicylic acid. * *p*-value ≤ 0.005.

**Table 3:** Adverse events during follow-up.

Adverse Events	Overall (n = 52)	Non-CAD Women (n = 23)	Women with CAD (n = 29)	*p*-Value
New coronary revascularization	4 (7.7)	1 (4.3)	3 (10.3)	0.420
Death	1 (1.9)	0 (0)	1 (3.4)	0.369
AMI	1 (1.9)	0 (0)	1 (3.4)	0.369
Stroke	1 (1.9)	0 (0)	1 (3.4)	0.369
MACE	7 (13.5)	1 (4.3)	6 (20.7)	0.086

CAD = coronary artery disease; AMI = acute myocardial infarction; MACE = major adverse cardiovascular events.

**Table 4:** Demographic and clinical characteristics of cases and matched controls (presence vs. absence MACE).

Characteristic	Overall (n = 52)	Non-MACE Women (n = 45)	Women with MACE (n = 7)	*p*-Value
Age, median (IQR)	35 (26–40)	35 (26–40)	35 (28–40)	0.213
Family History of CAD; n (%)	14 (26.9)	12 (26.7)	2 (28.6)	0.916
Previous EAC; n (%)	7 (13.5)	1 (2.2)	6 (85.7)	0.086
Follow-up time (years)	4.9 ± 2.3	5.0 ± 2.2	3.7 ± 2.6	0.157
Hospitalization days	6.9 ± 7.4	6.8 ± 7.9	7.3 ± 4.3	0.878
**Comorbidities; n (%)**				
Hypertension	9 (17.3)	8 (17.8)	1 (14.3)	0.820
Diabetes	1 (1.9)	0 (0)	1 (14.3)	0.010 **
Current smoker	40 (76.9)	34 (75.6)	6 (85.7)	0.553
BMI ≥ 30 kg/m^2^	14 (26.9)	12 (26.7)	2 (28.6)	0.916
Dyslipidaemia	16 (30.8)	12 (26.7)	4 (57.1)	0.104
Drug consumption	5 (9.6)	5 (11.1)	0 (0)	0.354
Depression	9 (17.3)	5 (11.1)	4 (57.1)	0.003 *
**Lipid levels and Atherogenic Indexes; n (%)**				
Total cholesterol	190.8 ± 50.6	190.6 ± 48.3	191.6 ± 67.3	0.064
HDL-C	45.8 ± 14.8	48.4 ± 14.5	32.6 ± 8.2	0.058
LDL-C	120.6 ± 46.2	122.2 ± 44.0	111.2 ± 61.6	0.141
Triglycerides	144.0 ± 117.6	125.9 ± 70.6	247.6 ± 242.5	0.001 *
Creatinine	0.75 ± 0.17	0.76 ± 0.16	0.72 ± 0.20	0.222
Glucose	105.2 ± 30.5	101.9 ± 24.9	124.6 ± 51.5	<0.001 *
Castelli Index	4.6 ± 1.8	4.3 ± 1.5	6.3 ± 2.5	0.007 *
Kannel Index	2.9 ± 1.4	2.7 ± 1.3	3.7 ± 2.2	0.006 *
TGL/HDL Index	3.8 ± 4.0	2.9 ± 2.0	8.1 ± 7.7	<0.001 *
Residual Cholesterol	26.8 ± 14.1	25.9 ± 13.7	32.0 ± 16.4	0.580
TGL/Glucose Index	8.71 ± 0.68	8.61 ± 0.56	9.26 ± 1.00	0.171
LVEF (%)	54.7 ± 11.7	54.9 ± 11.8	53.3 ± 11.6	0.738

MACE = major adverse cardiovascular events; IQR = interquartile range; CAD = coronary artery disease; BMI = body mass index; HDL = high-density lipoproteins; LDL = low-density lipoproteins; TGL = triglycerides. * *p*-value ≤ 0.005, ** As only one case had Diabetes, although the test identified the variable as statistically significant, It could be due to the limited sample size.

## Data Availability

The data used to support the findings of this study are available from the corresponding author upon reasonable request.
